# Animating fossilized invertebrates by motion reconstruction

**DOI:** 10.1093/nsr/nwad268

**Published:** 2023-10-14

**Authors:** Zixin Wang, Wei Zhang, Jiahao Li, Ji Wang, Yunqiang Yang, Tong Bao, Jianing Wu, Bo Wang

**Affiliations:** School of Advanced Manufacturing, Sun Yat-sen University, China; School of Engineering and Technology, China University of Geosciences (Beijing), China; Department of Mechanical Engineering, City University of Hong Kong, China; School of Aeronautics and Astronautics, Sun Yat-sen University, China; State Key Laboratory of Palaeobiology and Stratigraphy, Nanjing Institute of Geology and Palaeontology and Center for Excellence in Life and Paleoenvironment, Chinese Academy of Sciences, China; University of Chinese Academy of Sciences, China; School of Aeronautics and Astronautics, Sun Yat-sen University, China; School of Engineering and Technology, China University of Geosciences (Beijing), China; School of Ecology, Sun Yat-sen University, China; School of Advanced Manufacturing, Sun Yat-sen University, China; State Key Laboratory of Palaeobiology and Stratigraphy, Nanjing Institute of Geology and Palaeontology and Center for Excellence in Life and Paleoenvironment, Chinese Academy of Sciences, China

## Abstract

Taking the motion reconstruction of the Cretaceous hell ants as an example, this study shows how to achieve motion reconstruction in fossil invertebrates and discusses potential challenges and opportunities.

Fossils preserve valuable evolutionary traces of ancient species, waiting to be unveiled by scientists. Similarly to completing a jigsaw puzzle, scientists assemble the evolutionary history of extinct organisms by piecing together static anatomical features, dynamic motion clues or a combination of both. Compared with static information, dynamic information is more difficult to extract from photo-like fossils. This likely accounts for the prevailing bias toward anatomical interpretations in existing paleontological studies. However, merely relying on anatomical information might cause controversial hypotheses owing to the ambiguousness of motion features that play crucial roles in linking form and function. Sometimes, subtle to unnoticeable modifications in the morphology can modify kinematics and functions, and result in significant changes in behaviors, as suggested by functional morphology that describes the intricate interplay between the structural attributes and the behavioral and ecological traits of organisms. In recent years, the advancements in 3D digital technologies using computational methods have provided paleontologists with an unprecedented opportunity to non-destructively investigate the dynamic characteristics and mechanical performance of extinct species [1]. Motion reconstruction of extinct species can provide tangible and vivid understandings of their evolutionary traces and bring disappeared ecological landscapes back into our view [2,3]. For example, a study based on well-preserved skeletal fossils has examined the movement pattern and its influence on the feeding ecology of an extinct shark [4]. In addition, the locomotor characteristic of a Triassic theropod dinosaur has been reconstructed through musculoskeletal modeling and simulation [5].

Little attention, however, has been paid to reconstructing the motions of fossilized invertebrates, which is largely out of proportion to their unparalleled diversity on Earth and vital roles in ecosystems. Applying motion reconstruction techniques to invertebrates comes with more challenges than to vertebrates. Unlike skeletal and large-sized vertebrates, soft-bodied and small-sized invertebrates are less likely to fossilize and may possess more subtle motion clues that have not yet been uncovered [6]. To fill this gap, in this perspective, we present an approach for achieving motion reconstruction in fossil invertebrates and discuss potential challenges and future research directions. We also highlight the feasibility and potential of this approach to infer the functionality of structures of extinct species that lack modern counterparts by reconstructing the motion of the mandibles of the Cretaceous hell ants *Dhagnathos*.

Achieving motion reconstruction of fossilized invertebrates demands a joint endeavor of researchers from different disciplines, ranging from paleontology and biomechanics to mathematics and robotics. The workflow typically involves five key steps (Fig. 1): (i) collection of high-fidelity fossils through field research; (ii) 3D reconstruction of the targeted structure from high-fidelity fossils; (iii) detection and digitalization of hidden motion clues of the targeted structure; (iv) biomechanical rationalization and robot–physical validation of the examined motion characteristics; (v) inferring and restoration of the disappeared ancient ecology. Next, we will take our recent work on the motion reconstruction of the Cretaceous hell ants *Dhagnathos* as an example to illustrate how each step is carried out in detail.

**Figure 1.**
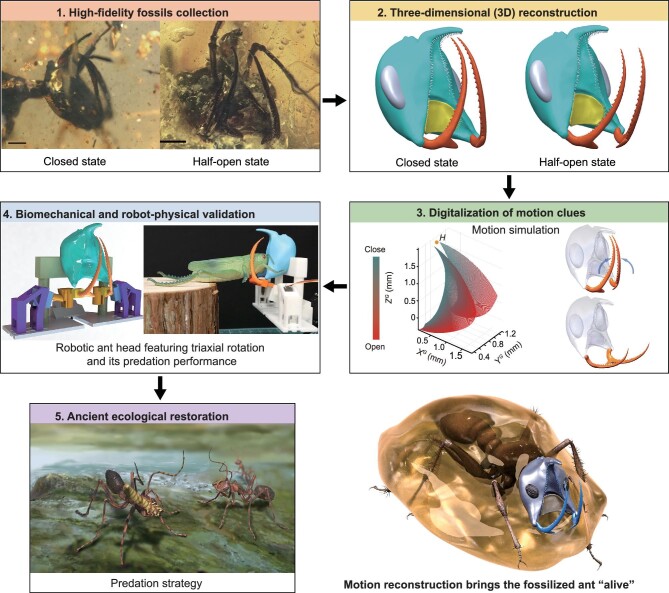
Flow chart of the basic steps of motion reconstruction of extinct invertebrate by taking the hell ant as an example. Scale bars: 500 μm.

The hell ants *Dhagnathos* are among the earliest ants on Earth and they possess distinctive morphology with movable mandibles and a unique horn lacking modern counterparts, both armed with sharp teeth [7]. Based on observed anatomical traits, the hell ant likely coordinates its mandibles and horn to create a clamping space for prey capture [7]. This unique way leads to an intuitive hypothesis that the hell ants may manipulate their mandibles dorsoventrally in the vertical plane for cooperation with the fixed horn. However, this hypothesis remains controversial owing to the lack of direct kinematic evidence [7]. Aiming to provide consolidating evidence for this debate, we conducted motion reconstruction on the mandibles of the hell ant *Dhagnathos* and found a new motion pattern unseen in ants (Fig. 1).

The first step in motion reconstruction is obtaining high-fidelity fossils through field research. High-fidelity fossils typically refer to fossils with minimal damage and alteration during fossilization, which can provide detailed information that is crucial to further scientific exploration. The two specimens of the hell ant *Dhagnathos* show little taphonomic distortions, from which we can extract sophisticated morphological information down to the tiny setae and mandible teeth (Supplementary Fig. 1A and Supplementary Fig. 4). According to stratigraphic data, the two amber specimens are from the same geological period, dating back to the Albian–Cenomanian boundary (∼100 million years ago), which allows us to put them together for comparative analysis [8].

Unlike most fossilized vertebrates manifested in 2D form in rocks, the majority of invertebrates in amber are preserved in 3D form, making it possible to reconstruct 3D morphologies with high fidelity. Realizing 3D reconstruction of the target structure of interest relies on a combination of photography and 3D modeling technology (Fig. 1). First, computed tomography (CT) scanning is used to extract morphological data from fossil specimens. The extracted data are then transformed into 3D models using 3D modeling software (e.g. Maya, Blender and others). The obtained 3D models allow operations such as zooming in and out, slicing, etc., so we can study the external and internal morphologies non-invasively. As a demonstration, the fossil ant specimens are first scanned layer by layer using micro-CT scanning techniques to acquire 3D morphology [7]. Based on these scanned images, we build the 3D models of the heads of the hell ants using Maya software (version: 2019, Autodesk, USA), in which their mandible positions can be quantified (Supplementary Fig. 5).

Detection and digitalization of motion cues concealed within fossils are huge challenges because extinct organisms, unlike living ones, cannot move (Fig. 1). When examining the kinematic properties of living creatures, video filming equipment can effectively capture the locomotion of the subject. The motion data can then be measured frame by frame by identifying key landmarks manually or automatically via specialized software. When it comes to the fossils, one alternative way is to collect multiple fossil individuals with target structures in different spatial positions, put them together and interpret them as a time series of movements, as if the extinct animals are really moving. For example, the mandibles of two fossilized ants are in different spatial positions—one in the closed state and the other in the half-open state (Supplementary Fig. 1A and Supplementary Movie 1), enabling us to obtain the motion behavior by comparing them. By introducing a 3D coordinate system, we quantify the mandible positions of the two specimens and find that the mandible position changes in three orthogonal planes simultaneously (Supplementary Fig. 1B). Such position changes cannot be achieved by the previously hypothesized dorsoventral rotation pattern and we rationally assume that the hell ant mandibles likely adopt a triaxial rotation pattern (Supplementary Movie 2). Because the mandible motion is limited to a fixed axis, the position changes between the three planes follow a linear relationship, by which the axis orientation can be determined for further mechanistic rationalization (Supplementary Fig. 1C).

Rationalizing the assumed kinematics can resort to biomechanical and robot–physical validation (Fig. 1). Taking into account the anatomical and kinematical constraints, biomechanical models that approximate how the extinct organisms move can be developed [1]. Considering the two assumed rotation patterns in the biomechanical model of the hell ant, the real-time spatial configurations of the mandibles when opening can be predicted. The theoretical results show that, compared with the dorsoventral rotation, the triaxial rotation significantly expands the clamping space of the mandibles, which may benefit the predation behavior of the hell ants (Supplementary Fig. 2C). To further validate the predation performance for the two rotation patterns, we animate the fossilized ants by constructing two robotic models of the hell ants that feature triaxial or dorsoventral rotation using 3D printing technology. Meanwhile, various arthropod models are used as potential prey to mimic real-world predation behaviors. The robotic model that dorsoventrally manipulates its mandibles suffers from insufficient vertical operation space underneath these prey bodies and fails to grip prey firmly (Supplementary Movie 3). By contrast, the robotic ant head with triaxial rotation frees up the need for vertical space by decomposing motion into other planes and thus successfully constrains these prey by using its mandibles and horn in terrestrial scenarios (Supplementary Fig. 2D and Supplementary Movie 4). As a result, the triaxial rotation is more suitable for the terrestrial invertebrate *Dhagnathos* to fully exploit the coordination between mandibles and horns for predation with a high success rate, while the dorsoventral rotation pattern can be a powerful way for predators living in aquatic or aerial environments with ample vertical operation space, as observed in some documented aquatic or aerial species.

The results of motion reconstruction allow researchers to further infer and restore the relevant aspects of the ancient ecology of the extinct invertebrates (Fig. 1). The disappeared ecology can be visualized through collaborations between scientists and painters, further deepening our understanding of the ecology of ancient invertebrates. With the aid of a painter, the scene of the predation behavior of the hell ants *Dhagnathos* has been vividly restored (Fig. 1) and this tangible outcome could facilitate the penetration of paleontology for the public. Moreover, the revealed predation strategy of fossilized *Dhagnathos* based on triaxial rotation is different from that of other lineages of ancient hell ants and may result in past ecological niche diversification.

Despite the exciting contributions of motion reconstruction to appreciating the evolution and paleontology of extinct invertebrates, certain challenges and opportunities also arise. First, the 3D reconstruction of tiny invertebrates is vulnerable to accuracy loss, especially when dealing with sophisticated structures typically at the micro/nano scale [9]. The accuracy loss can be minimized from two stages: (i) increasing the in-plane resolution or reducing the layer spacing when scanning fossils layer by layer in the pre-data acquisition stage; (ii) introducing machine-learning-based algorithms that have been widely employed in image process scenarios could enhance the fidelity by removing noise and artifacts, and filling in missing information in the post-data processing stage [10]. Second, performing robot–physical validation calls for concerted efforts from paleontologists, bio-mechanists and roboticists to scientifically smoothen the paleontology–robot transformation. Bio-mechanists are expected to rationally predict functionally related biological elements and determine whether a scaling effect is involved. If so, roboticists have to 1:1 replicate the robot models of biological counterparts. However, 1:1 replication remains challenging even for state-of-the-art robotics, mainly due to the bulkiness of actuators compared with the invertebrate microstructures. Miniaturizing robotics can resort to the use of muscle-like actuators including dielectric elastomer actuators, shape-memory alloys or responsive materials. Furthermore, finite-element analysis that can analyse the mechanical properties of complex structures by subdividing them into a finite number of elements of simple geometry serves as a powerful tool to computationally simulate 3D models of biological structures [11,12].

In conclusion, the proposed holistic approach to animating fossilized invertebrates through motion construction has been described by using the hell ant as an example. Providing kinematic insights into the ancient ecology by robotic demonstration, such an approach serves as a catalyst for the combination of paleontology and other disciplines and open doors for multi-dimensionally understanding invertebrate paleontology.

## Supplementary Material

nwad268_Supplemental_Files
