# Supplementary material for: Animating fossilized invertebrates by motion reconstruction
Source: Natl Sci Rev. 2023 Oct 14;10(12):nwad268. doi: 10.1093/nsr/nwad268 (PMC10684265; doi:10.1093/nsr/nwad268)
Supplement: nwad268_Supplemental_Files [file nwad268_supplemental_files.zip › Supplementary Information.docx]

Supplementary information for

**Animating fossilized invertebrates by motion reconstruction**

Zixin Wang†, Wei Zhang†, Jiahao Li, Ji Wang, Yunqiang Yang,

Tong Bao*, Jianing Wu*, Bo Wang*

† These two authors contributed equally to this work.

*Corresponding author.

Email: baot3@mail.sysu.edu.cn (T.B.)

wujn27@mail.sysu.edu.cn (J.N.W.)

bowang@nigpas.ac.cn (B.W.)

**This PDF file includes:**

Triaxial rotation of the mandibles

Expanded clamping space

Supplementary Methods

Supplementary Fig. 1. Mandible motion extraction of the mandibles of the hell ants from fossils.

Supplementary Fig. 2. Expanded clamping space.

Supplementary Fig. 3. Measurements of the mandible spatial configuration in closed and half-open states.

Supplementary Fig. 4. Photomicrographs of two amber specimens of *Dhagnathos autokrator*.

Supplementary Fig. 5. Three-dimensional models of the mandibles of *D. autokrator*.

Supplementary Table 1. The spatial configurations of the mandible in closed and half-open states.

Supplementary Table 2. Details on the respective condition of the two specimens

Supplementary Table 3. Systematic paleontology of *D. autokrator*

**Other Supplementary Materials for this manuscript include the following:**

Supplementary Movies S1 to S4

Supplementary Data 1 to 2

**Triaxial rotation of the mandibles**

The main principle for deciphering motion cues from static fossil specimens is to equate spatial configuration changes between two fossil individuals in different states to a temporal sequence performed by a live individual. Toward this, we first three-dimensionally reconstruct two high-fidelity fossils of *D. autokrator* with mandibles in closed and half-open states, and comparatively characterize the spatial configurations of their mandibles (see Supplementary Methods details). These fossil specimens were previously studied and analyzed by Perrichot et al. [1]. For better illustration, we introduce a global coordinate system *G*, where the origin ***O*** is fixed to the right mandible, the *XG*-axis aligns with the longitudinal axis of the ant body, the *YG*-axis is in the horizontal plane and perpendicular to the *XG*-axis, and the *ZG*-axis is determined according to the right-handed rule (see Supplementary Methods details).

In the closed state, the ant mandibles are brought together, with their tips almost contacting each other (Supplementary Fig. 1A and Supplementary Movie 1). As transitioning into the half-open state, the mandibles show notable changes in the spatial configurations, characterized by the differences in the intermandibular distance *w* in the *XGOYG* plane, mandibles-to-horn distance *h* in the *XGOZG* plane, and mandibular postures (Supplementary Fig. 1A and Supplementary Movie 1). These concurrent three-plane changes cannot be achieved via the previously assumed dorsoventral motion in a single plane [2]. As a result, we hypothesize that this ant may utilize a more flexible yet unknown rotation pattern that operates the mandibles in multiple planes.

We then test this hypothesis by quantifying the spatial configuration changes to determine the mandible motion characteristics. For simplicity and considering the symmetry between the pair of mandibles, we focus on the right mandible for further analysis. The mandible spatial configuration can be quantitatively described by introducing three angles, namely *α*, *β*, and *γ*, corresponding to the angle relative to the *XG*-axis, *YG*-axis, and *ZG*-axis, respectively (Supplementary Fig. 1A). We measure these angles in the closed and half-open states of the mandibles (Supplementary Table 1) and find that all of them changed between the two different states. This indicates that the ant mandibles may simultaneously move in three orthogonal planes, aptly termed triaxial rotation, distinct from the previously assumed dorsoventral rotation in the single vertical plane [1].

To further examine this unique motion, we calculate the respective changes in these rotation angles, defined by *∆α*, *∆β*, and *∆γ* (Supplementary Fig. 1B). Because the ant mandible motion is confined to a fixed pivot [3], the changes in the three angles should follow linear relationships, namely. *∆β*=*k*1*∆α* and *∆γ*=*k*2*∆α*, in which and through fitting experimental data (see Supplementary Methods details). The revealed linear relationships allow us to locate the spatial position of the pivot for triaxial rotation. As shown in Supplementary Fig. 1C, the pivot makes an angle and with respect to the positive *ZG*-axis and *XG*-axis, respectively (see Supplementary Methods details).

We next visualize the key events of the mandible motion for the dorsoventral and triaxial rotation and compared them with the experimental observations (Supplementary Fig. 1D and Supplementary Movie 2). Only the mandible-to-horn distance changes for the dorsoventral rotation, leaving the mandibular distance and the mandible posture unchanged. While for the triaxial rotation, all three features are in a way that aligns with the observations from fossil specimens, validating our hypothesis.

**Expanded clamping space for triaxial rotation**

To dig out the essential role of this unique kinematics in the mandible functionality, we built a biomechanical model to probe whether the unique triaxial rotation can benefit the predatory performance of the hell ants. One of the important factors for evaluating predatory performance is clamping space [3], which is closely related to the size of prey that can be captured. Given the joint role of the movable mandibles and the fixed horn in predation, the clamping space is defined as the area they create (Supplementary Fig. 1A). As a result, the dimensions of the clamping space, namely the width (*w*) and height (*h*), are characterized by the intermandibular distance and the mandibles-to-horn distance, respectively. Since the horn tip *H* always keeps still, the clamping space is mainly dictated by the spatial configurations of the moveable mandibles. Incorporating the triaxial rotation into our biomechanical model allowed to predict the real-time spatial configurations of the mandibles when opening. As illustrated in Supplementary Fig. 1C, given a rotation angle τ about the rotation axis *e*, the mandible tip moves from its initial position to a new position, which can be determined using the biomechanical model (see Supplementary Materials) [4, 5].

To unveil the potential advantages of the triaxial rotation, we plot the mandibles’ profiles when opening via dorsoventral rotation and triaxial rotation in Supplementary Fig. 2A (Supplementary Movie 2). The latter is found to generate a larger area between the mandibles and the horn. This difference can be explained by depicting the trajectories of the mandible tip for the two rotation patterns. As shown in Supplementary Fig. 2B, we find no significant difference in the position changes of the mandible tip between the two rotation patterns, except in the *YG*-axis direction associated with the width. As a result, the clamping space for triaxial rotation is nearly as high as the dorsoventral rotation but 4.6 times wider (Supplementary Fig. 2C). It suggests that the triaxial rotation provides expanded clamping space for the ant, making capturing a broader range of prey possible.

The predation performance is further validated by developing robotic models of the ant head at a magnification of 40 x (Supplementary Fig. 2D). The robotic ant head can perform triaxial rotation via spatially inclined pivots under motor actuation. A control sample that rotates dorsoventrally via perpendicular pivots is also fabricated for comparison. We challenge the two robotic ant heads with various arthropods made of rubber to mimic real-world predation behaviors. The robotic ant head featuring triaxial rotation successfully captures a variety of rubbery arthropods by first gripping them between mandibles and then constraining them in the clamping space formed by the toothed mandibles and horn (Supplementary Fig. 2D and Supplementary Movie 3). In contrast, the control sample fails to do so due to the limited clamping space (Supplementary Movie 4). Collectively, triaxial rotation may be the right way for *Dhagnathos* to flexibly manipulate their mandibles to fully exploit the coordination between mandibles and horns for successful predation.

# Supplementary Methods

**Examination and imaging of fossils**

We conducted a comprehensive re-examination of two specimens of *D. autokrator*, featuring closed and half-open mandibles, which originate from mid-Cretaceous Kachin amber [6] (Supplementary Fig. 1A, B, and Supplementary Fig. 4). These specimens were previously described by Perrichot et al. [1], and the radiometric dating of zircons obtained from the amber-bearing bed has yielded a maximum age of 98.79±0.62 Ma [7]. This age aligned with the late Albian to early Cenomanian interval within the mid-Cretaceous period. It was reasonable to assume that the age of the amber material itself does not significantly deviate from this timeframe [8]. Consequently, the two amber specimens examined in this study could be assigned to the same geological period (Supplementary Table 2). Furthermore, it was worth noting that the two examined amber fossils of hell ants presented the mandibles in distinct positions, a serendipitous circumstance that allowed for comprehensive determination of mandible positions through an analysis of images captured from various angles. The morphological characteristics of the hell ant model had been thoroughly scrutinized in a prior publication by Perrichot et al [1] (Supplementary Table 3).

Specifically, specimen HA03, representing *D. autokrator*, exhibited closed mandibles and displayed a near-complete preservation despite minor lateral distortion (Supplementary Fig. 1A, Supplementary Tables 2 and 3, Supplementary Fig. 4A). On the other hand, specimen IGR.BU-003, characterized by well preservation, showcased mandibles in a half-open state and exhibited no evidence of compression (Supplementary Fig. 1B, Supplementary Tables 2 and 3, Supplementary Fig. 4B). Previous investigation of these amber fossils provided substantial evidence to confidently exclude the possibility of distortion [1]. This conclusion was based on careful assessments of the overall shape and the characteristic flow texture of the amber, further affirming the integrity of these specimens under scrutiny. The fossil specimens were examined and photographed dry using a stereomicroscope system (ZEISS Stereo Discovery V16) at the Nanjing Institute of Geology and Palaeontology, Chinese Academy of Sciences. All images were taken by employing digitally stacked photomicrographic composites of approximately 40 individual focal planes using the image editing software Helicon Focus 6 (HeliconSoft, Ukraine) to create an extended field of depth image and perform further three-dimensional reconstructions.

**Three-dimensional reconstructions**

We reconstructed the spatial configurations of their head (Supplementary Fig. 5), with particular emphasis on their mandibles in closed (Supplementary Fig. 5A, Supplementary data 1) and half-open states (Supplementary Fig. 5B, Supplementary data 2), using Maya software (version: 2019, Autodesk, USA) based on the existing photomicrographs of specimens and the three-dimensional models constructed by Barden et al [2]. The two 3D models of the ant heads have been provided, facilitating access for reference and further analysis. The datasets supporting this article have been uploaded as part of the supplementary material.

**Characterization of mandible kinematics**

We introduced a coordinate system, where the origin *O* sited at the connection joint, *XG*-axis was aligned with the long axis of the ant head, *ZG*-axis was perpendicular to the horizontal plane, and *YG*-axis was determined by the right-handed system to quantify the morphology of the ant mandibles and horn (Supplementary Fig. 3A). Due to the structural symmetry, we focused on the profile of the right mandible and the right half of the horn. We examined the changes in mandible configurations between the closed and half-open states to quantify the mandible motion. We introduced three parameters, *αi*, *βi* and *γi*, to denote the angle of the mandibles concerning the *XG*-, *YG*-, and *ZG*-axes, respectively. It should be noted that the subscript *i* (*i* = 1 or 2) denoted closed and opened states, respectively. Then the three angles were measured from three orthogonal views in the two states (Supplementary Fig. 3A and B). Thus, the respective differences in these angles between the two states, referred to *∆αi*, *∆βi* and *∆γi*,could be calculated by , , and . At *i* = 1 for the initially closed state, we arrive at *∆α1*=*∆β1*=*∆γ1*=0. The spatial location of the axis ***e*** could be described by introducing two angles, and , denoting the directions related to the *ZG*-axis and *XG*-axis, respectively. As a result, the vector of the axis ***e*** was , in which , and .

**Analytical modeling for calculating clamping space of the hell ants**

To describe the mandible profile, we first uniformly measured the coordinates of 200 points along the mandible in the software Solidworks (Dassault Systèmes, France), and fitted them using a polynomial function. We then build an analytical model to predict the real-time spatial profiles of the mandible when opening. Taking the tip of the mandible as an example, given a rotation angle τ about the inclined axis *e*, the mandible tip would move from its initial position to a new position . According to the coordinate transformation, we could determine the new position using with [9], where **I** was a third order identity matrix and was the skew-symmetric cross-product operator defined by:

|  |  | (1) |
| --- | --- | --- |

The horn tip *H* always kept still at . As a result, the real-time width *w* and height *h* of the clamping space when the mandibles open could be calculated by and .

**Design and fabrication of robotic models of the hell ant head**

To validate the predation performance, robotic models of the hell ant head were designed at a 40× magnification of the biological counterpart and fabricated using a 3D printer (Formlabs, USA) with photopolymer (RS-F2-GPWH-04). The photopolymer featured high rigidity and strength, similar to the material properties of the strongly sclerotized cuticle in the ant mandibles. We installed the robotic ant head on a robotic arm (Robotic arm JIBOT1, Zhongling Technology, China). The 3D models of the mandibles were actuated by using two servo motors (MG-90, HexTronics Ltd., Kowloon Bay, Hong Kong) with a peak power of 1.9 W and a maximum rotational speed of 9.52 rad/s. The artificial mandible models were controlled by an Arduino Uno R2 Board (Arduino.cc, Germany), and the robot arm was powered by a 7.4 V and 3.0 A power adaptor (Zhongling Technology, China). The clamping objects were rubbery models of locusts, unicorns, crickets, weevils, and spiders (see Supplementary Movies S3 and S4).

**Supplementary Figures**

| 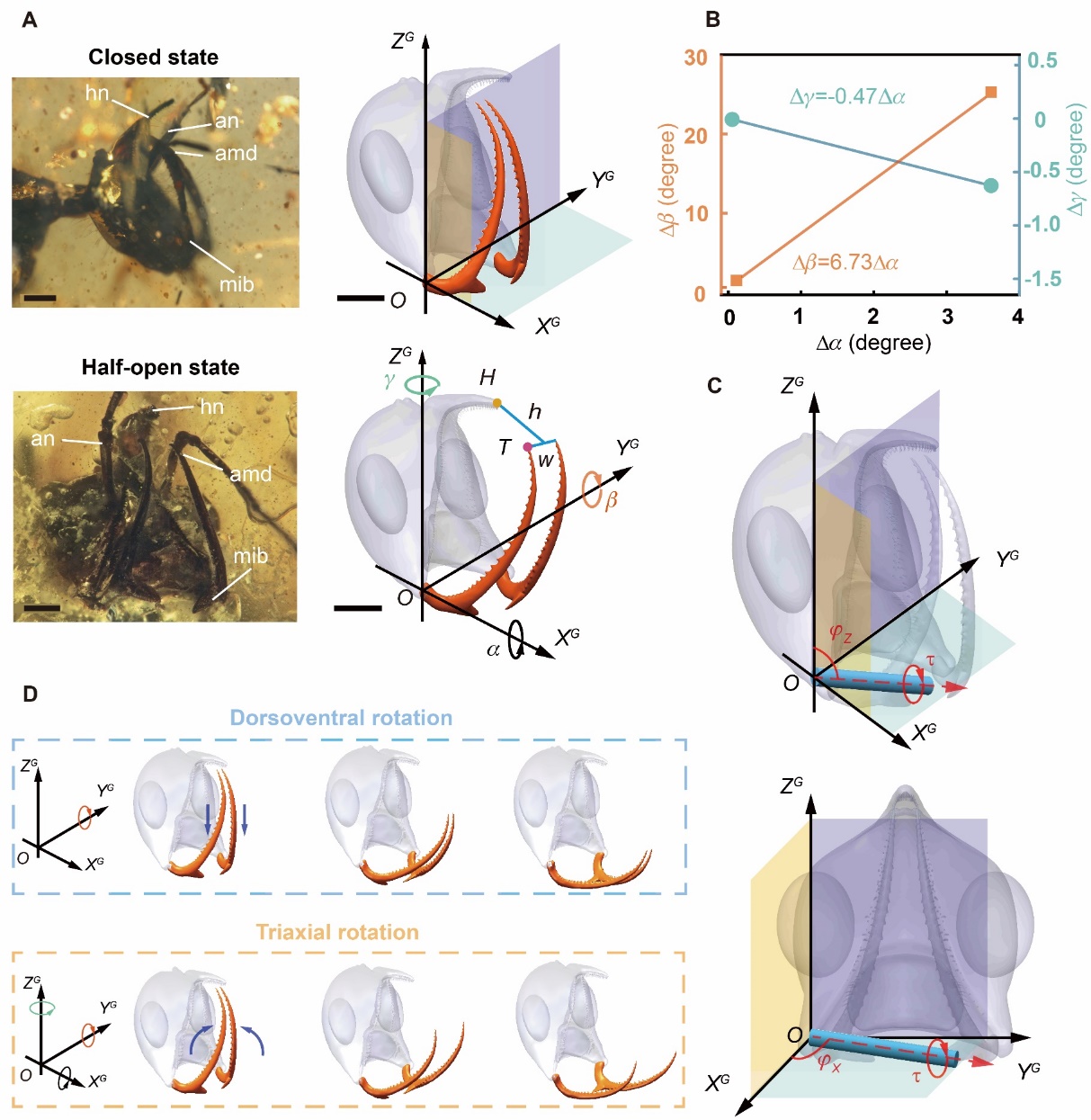 |
| --- |
| **Supplementary Fig. 1. Mandible motion extraction of the mandibles of the hell ants from fossils.** (A)Photomicrograph and 3D model ofthe mandibles of *D. autokrator* in the closed statefrom mid-Cretaceous Kachin amber, HA03, female (upper). Photomicrograph and three-dimensional model of the mandibles of *D. autokrator* in the half-open state from mid-Cretaceous Kachin amber, IGR.BU-003, female (lower). Schematics of clamping space formed by the mandibles and the horn in the coordinate *G*, characterized by width *w* and height *h*. The orange point *H* and the purple point *T* denote the tips of the horn and mandible, respectively. Schematics of possible movements of the mandibles, including rotating by *α* around the *XG*-axis, by *β* around the *YG*-axis, and by *γ* around the *ZG*-axis. Scale bars: 500 μm. (B) Relationships between *∆β* and *∆α* as well as *∆γ* and *∆α*. (C) Schematics of triaxial rotation around a spatially inclined axis ***e***, which made an angle of *φz* to the *ZG*-axis and an angle of *φx* to the *XG*-axis. *τ* represents the rotation angle. (D) Motion simulation of the mandible opening process from the oblique view for the dorsoventral (upper) and triaxial (lower) rotation patterns. amd, apical portion of *D. autokrator* mandibles; an, antenna of *D. autokrator*; mib, mandibular medioventral blade; ho, horn of *D. autokrator*. |

| 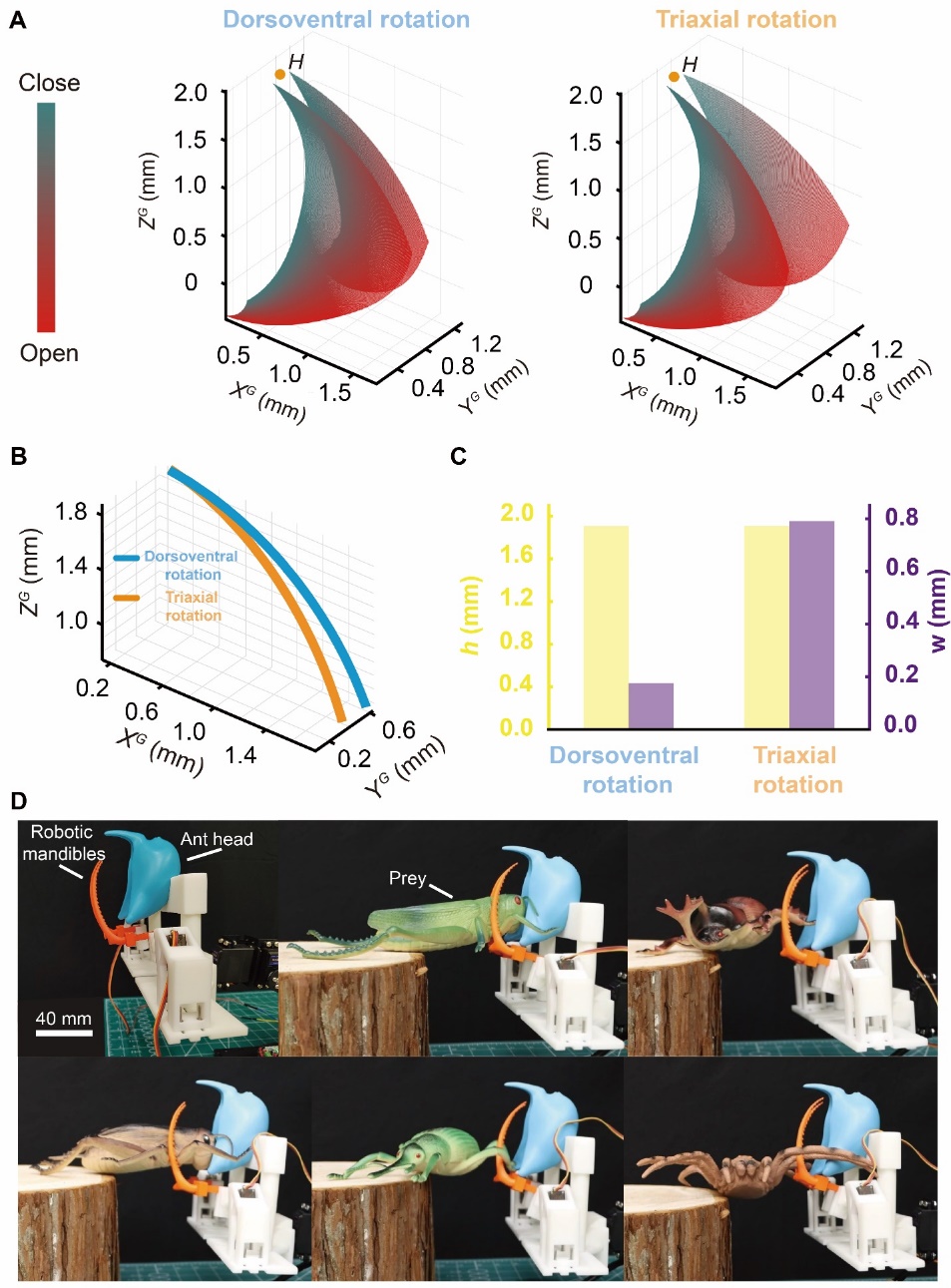 |
| --- |
| **Supplementary Fig. 2. Expanded clamping space of the hell ants using triaxial rotation.** (A) Real-time spatial configurations of mandibles for the dorsoventral (left) and triaxial (right) rotation patterns. The color changed from blue to red, indicating that the mandibles opened gradually from the closed position. (B) Trajectories of the mandible tip for the two rotation patterns. (C) The height *h* and width *w* of the clamping space of the ants via various rotational patterns. Yellow: *h*; Purple: *w*. (D) Robotic ant head featuring triaxial rotation and its predation performance. |

| 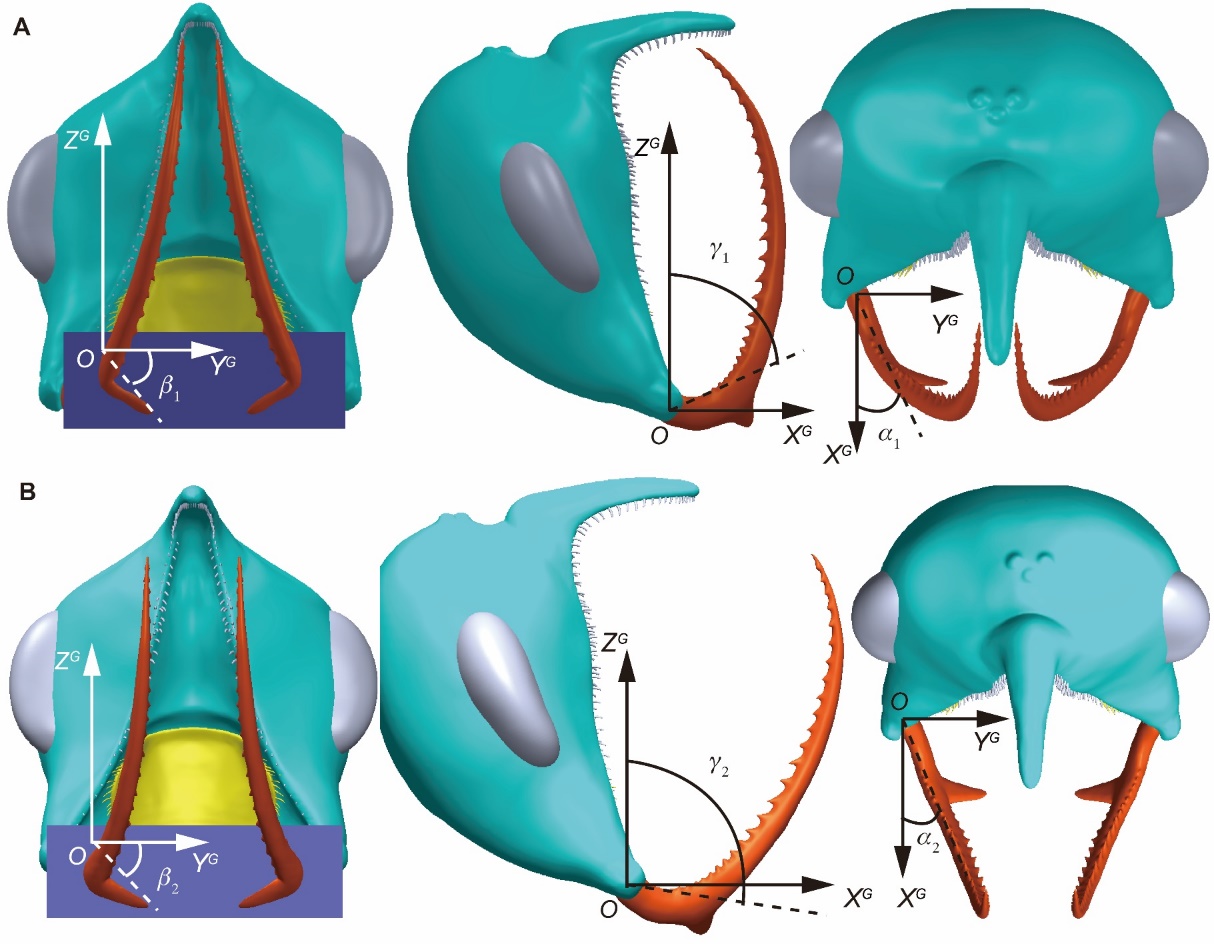 |
| --- |
| **Supplementary Fig. 3. Measurements of the spatial configurations of the mandibles in closed and half-open states.** The frontal, lateral, and dorsal views of the ant head were shown from left to right when the mandibles were closed (A) and half-open (B), respectively. Here , , and (*i*=1 for closed state or 2 for half-open state) denote the angles of the right mandible with respect to the *XG*-axis, *YG*-axis, and *ZG*-axis, respectively. |

| 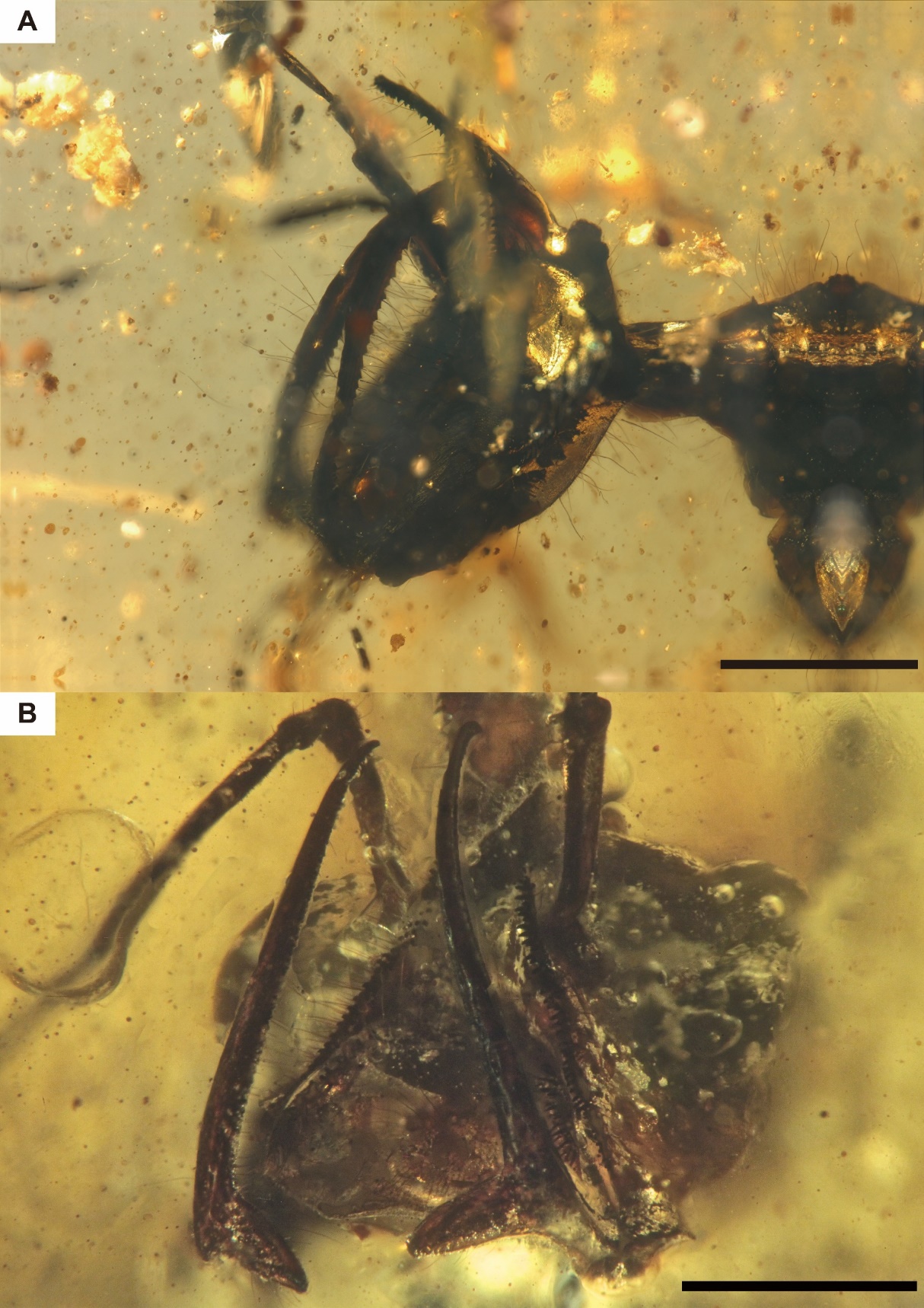 |
| --- |
| **Supplementary Fig. 4.** **Photomicrographs of two amber specimens of *Dhagnathos autokrator*.** (A) Specimen HA03. Scale bar: 1 mm, (B) holotype IGR.BU-003, Scale bar: 1 mm. |

| **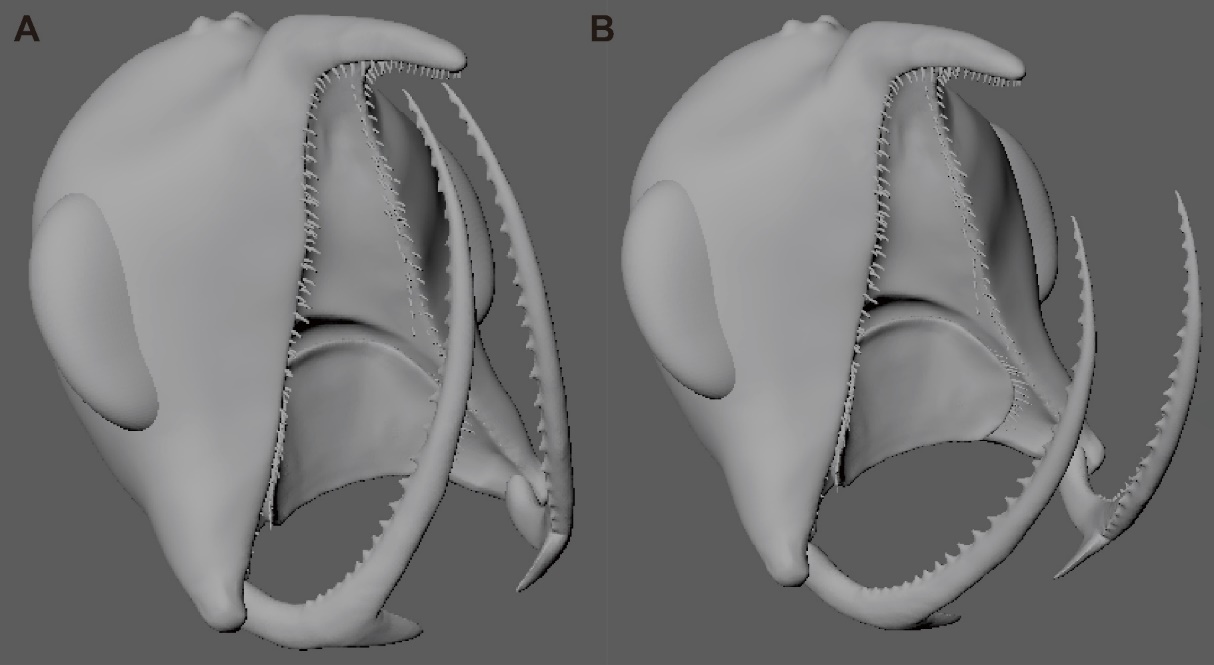** |
| --- |
| **Supplementary Fig. 5. Three-dimensional models of the mandibles of *D. autokrator* in the closed (A) and half-open state (B) reconstructed in Maya software (version: 2019, Autodesk, USA)** |

**Supplementary Tables**

**Supplementary Table 1.** **The spatial configurations of the mandible in closed and half-open states.**

| Positions | *α* (degree) | *β* (degree) | *γ* (degree) |
| --- | --- | --- | --- |
| Closed | -50.69 | 74.90 | 24 |
| Half-open | -47.04 | 98.86 | 22.3 |

**Supplementary Table 2. Details on the respective condition of the two specimens [1]**

| **Specimen** | **Repository** | **Age of the specimens** |
| --- | --- | --- |
| **IGR.BU-003** | Geology Department and Museum of the University Rennes 1, France | The radiometric dating of zircons obtained from the amber-bearing bed has yielded a maximum age of **98.79±0.62 Ma**. This age aligns with the late Albian to early Cenomanian interval within the mid-Cretaceous period. It is reasonable to assume that the age of the amber material itself does not significantly deviate from this timeframe. **Consequently, the two amber specimens examined in this study can be assigned to the same geological period.** |
| **HA03** | Huangyiren Amber Museum, Taiwan |

**Supplementary Table 3. Systematic paleontology of *D. autokrator* [1]**

| **Systematic paleontology** | **Detail information** |
| --- | --- |
| **Etymology.** | The specific epithet is derived from ‘autokrator’ (Greek), which signifies a self-ruling individual with absolute power, not bound by superiors. This term is used to highlight the immensely powerful nature of this ant species. |
| **Holotype.** | **IGR.BU-003, alate female.** |
| **Additional specimens** | **HA03,** XA01 and RM1, three alate females. |
| **Horizon and locality.** | **Upper Cretaceous, upper Albianelower Cenomanian (ca. 99 Ma)**; **in amber from the Hukawng Valley, Kachin State, Myanmar.** |
| **Diagnosis.** | **As for the genus, by monotypy.** |
| **Description(gyne).** | Body length ca. 14 mm. The cuticle is typically smooth, lacking any noticeable sculpturing. It is sparsely adorned with thin, long, upright setae. Furthermore, the head is densely populated with short, pressed-down setae on both the vertex and genae. |

# Supplementary Movie 1. 3D model of the mandibles in the fully closed or half-open states

# Supplementary Movie 2. Dorsoventral and triaxial rotation of the mandibles

# Supplementary Movie 3. The dorsoventral rotating robotic ant head fails to capture prey.

# Supplementary Movie 4. The robotic ant head featuring triaxial rotation successfully captures prey

# Supplementary Data 1. 3D model of the mandibles of *D. autokrator* in the closed state

# Supplementary Data 2. 3D model of half-open mandibles of *D. autokrator*.

# References

1. Perrichot V, Wang B, Barden P. New remarkable hell ants (Formicidae: Haidomyrmecinae stat. nov.) from mid-Cretaceous amber of northern Myanmar. *Cretac Res*. 2020; **109**: 104381. doi: 10.1016/j.cretres.2020.104381

2. Barden P, Perrichot V, Wang B. Specialized Predation Drives Aberrant Morphological Integration and Diversity in the Earliest Ants. *Curr Biol*. 2020; **30**(19): 3818-3824. doi: 10.1016/j.cub.2020.06.106

3. Zhang W, Wu Z, Wang Z *et al.* Double-rowed teeth: design specialization of the H. venator ants for enhanced tribological stability. *Bioinspir Biomim*. 2021; **16**(5): 055003. doi: 10.1088/1748-3190/ac124a

4. Zhang W, Li M, Zheng G *et al.* Multifunctional mandibles of ants: Variation in gripping behavior facilitated by specific microstructures and kinematics. *J Insect Physiol*. 2020; **120**: 103993. doi: 10.1016/j.jinsphys.2019.103993

5. Zhang W, He Z, Sun Y *et al.* A Mathematical Modeling Method Elucidating the Integrated Gripping Performance of Ant Mandibles and Bio-inspired Grippers. *J Bionic Eng*. 2020; **17**(4): 732-746. doi: 10.1007/s42235-020-0065-9

6. Yu T, Kelly R, Mu L *et al.* An ammonite trapped in Burmese amber. *Proc Natl Acad Sci USA*. 2019; **116**(23): 11345-11350. doi: 10.1073/pnas.1821292116

7. Shi G, Grimaldi DA, Harlow GE *et al.* Age constraint on Burmese amber based on U–Pb dating of zircons. *Cretac Res*. 2012; **37**: 155-163. doi: 10.1016/j.cretres.2012.03.014

8. Brookes MJ, Woolrich M, Luckhoo H *et al.* Investigating the electrophysiological basis of resting state networks using magnetoencephalography. *Proc Natl Acad Sci USA*. 2011; **108**(40): 16783-16788. doi: 10.1073/pnas.1112685108

9. Giulietti F, Tortora P. Optimal rotation angle about a nonnominal Euler axis. *J Guid Control Dyn*. 2007; **30**(5): 1561-1563. doi: 10.2514/1.31547
